# Pluripotent Stem Cells: Current Understanding and Future Directions

**DOI:** 10.1155/2016/9451492

**Published:** 2015-12-20

**Authors:** Antonio Romito, Gilda Cobellis

**Affiliations:** ^1^Centogene, Schillingallee 68, 18057 Rostock, Germany; ^2^Department of Biophysics, Biochemistry and General Pathology, Second University of Naples, Via L. de Crecchio 7, 80131 Napoli, Italy

## Abstract

Pluripotent stem cells have the ability to undergo self-renewal and to give rise to all cells of the tissues of the body. However, this definition has been recently complicated by the existence of distinct cellular states that display these features. Here, we provide a detailed overview of the family of pluripotent cell lines derived from early mouse and human embryos and compare them with induced pluripotent stem cells. Shared and distinct features of these cells are reported as additional hallmark of pluripotency, offering a comprehensive scenario of pluripotent stem cells.

## 1. Introduction

The first evidence suggesting the existence of “special cells,” today known as stem cells, able to self-renew and to differentiate into specialized cell types, dates back in 1961 when two scientists, Drs. James A. Till, a biophysicist, and, Ernest A. McCulloch, a hematologist, accidentally observed that the intravenous injection of bone marrow cells in previously irradiated mice led to the formation of colonies of proliferating cells in the spleen of those animals. The injected cells were blood-forming progenitor cells, able to fully regenerate the blood cells, and opening toward the clinical use of bone marrow transplantation for haematopoietic disorders [1]. Since then, the following works have reported the isolation, identification, and characterization of different types of stem cells.

Today, the field of stem cell research is in a rapid and dynamic expansion, representing one of the most exciting areas in life science. The importance of studying the biology of stem cells relies in their wide range of applications. In basic research, stem cells represent a powerful system to study gene function and the physiological processes occurring during development. In biomedical research, stem cells are used to study the pathogenesis of human genetic disease, to identify new diagnostic and prognostic biomarkers, and to test improved drugs. However, what renders stem cell research extremely important is the vast potential of clinical applications of these cells. Their capacity to differentiate into specific cell types could be used in regenerative medicine to treat damaged or diseased tissues through cell-replacement therapies. Recently, the US Food and Drug Administration (FDA) approved clinical trials using stem cells for the treatment of heart disease [2]. Although some stem cell therapies are in clinical trials, a lot more basic research is needed before therapies using differentiated stem cell-derivatives can be applied in humans.

Over the last years, the major advances and discoveries in stem cell research have been made in pluripotent stem cells (PSCs). The definition of pluripotent stem cell is based on two properties: self-renewal and potency. The self-renewal is the capacity of the stem cells to divide indefinitely, producing unaltered cell daughters maintaining the same properties of the progenitor cell. In particular conditions or under specific signals, a stem cell is able to exit from self-renewal and engage a program leading to differentiate into specialized cell types deriving from the three germ layers (ectoderm, endoderm, and mesoderm) [3].

There are two types of PSCs, embryonic stem cells (ESCs) and induced pluripotent stem cells (iPSCs). ESCs are derived from the inner cell mass (ICM) of preimplantation embryos [4, 5] and can be indefinitely maintained and expanded in the pluripotent state* in vitro*. Pluripotent stem cells can also be obtained by inducing dedifferentiation of adult somatic cells through a recently developed* in vitro* technology, known as cell reprogramming [6, 7]. Similarly to ES, iPS cells can be expanded indefinitely and they are capable to differentiate in all the derivatives of the three germ layers.

The aim of this review is to provide a detailed overview of the recent discoveries in ESC and iPSC research. We will compare murine and human ESCs, highlighting common and distinct features of pluripotency. Particularly, we will discuss the current notion of “ground state” of pluripotency for mESCs and whether such a naïve state can exist for hESCs. Furthermore, we will review the most recent advances in iPSCs and point out some key hurdles in cell reprogramming. Finally, we will discuss the potential applications of pluripotent stem cells, with a special emphasis on iPSCs as promising and exciting source to model human diseases and to develop cell-based therapies.

## 2. Embryonic Stem Cells

### 2.1. Mouse Embryonic Stem Cells: The “Ground State” of Pluripotency

**Figure 1:**
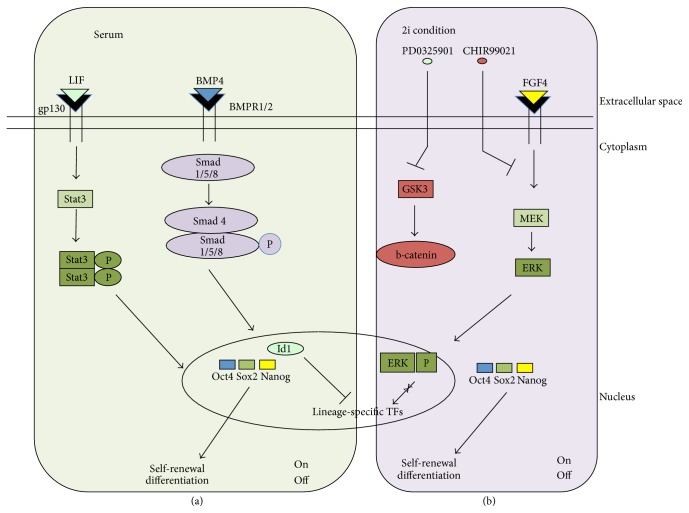
Signal transduction pathways in serum- and 2i-cultured mESCs.

Murine ESCs (mESCs) were first isolated in 1981 from the ICM of mouse blastocyst, the part that will give rise to the embryo. They can be maintained indefinitely in culture through self-renewing division and, more importantly, are pluripotent, retaining the ability to differentiate into all somatic cell lineages [8]. mESCs were originally established and maintained in presence of serum on mouse embryonic fibroblasts (MEFs) as feeder cells, growing as round-shaped colonies of tightly packed cells, suggesting close cell membrane contacts and inability to “walk” on the plate. Maintaining of the self-renewing state of mESCs can be obtained by adding the cytokine leukaemia inhibitory factor (LIF) in culture medium [9]. The LIF/gp130 receptor interaction on the cell surface activates Stat3, the downstream effector of multiple intracellular pathways, including JAK/Stat3, PI3K/Akt, and MAP/ERK [10]. In response to the activation of these pathways, Stat3 is phosphorylated and forms homo- or heterodimers that translocate from the cytoplasm to nucleus, where it binds to specific DNA elements activating the transcription of pluripotency factors (Figure 1(a)) [11]. In an attempt to find molecules able to guarantee the self-renewal, the transcription factor CP2-like 1 (Tfcp2l1) was identified as the target of the LIF/Stat3-mediated pathway controlling mESC self-renewal, and forced expression of Tfcp2l1 was shown to recapitulate the self-renewal-promoting effect of LIF [12].

However, LIF alone is not sufficient to maintain mESC self-renewal, as the cells also require the presence of foetal calf serum. Bone morphogenetic proteins (BMPs), members of the transforming growth factor (TGF)-*β* family, present in the serum, act in conjunction with LIF, enhancing the self-renewal and pluripotency of mESCs [11, 13]. The binding of BMP4 to its receptors (BMPR1/2) triggers phosphorylation of Smad proteins (Smad1, Smad5, and Smad8). Once phosphorylated, they form a complex with Smad4 and translocate into the nucleus [14], where they activate expression of inhibitor of differentiation (Id) gene 1 (Id-1), critical for suppressing ESC differentiation and sustaining pluripotency [11]. Overexpression of Id1 led mESCs to self-renew in absence of BMP4 and its disruption led to decreased Nanog expression and mESCs failed to maintain self-renewal [15] (Figure 1(a)).

Hence, LIF and BMP signalling pathways play a central role in maintaining the pluripotent phenotype. In absence of any supplements to the culture medium, mESCs tend to lose their pluripotency and self-renewing capacity due to fibroblast growth factor 4 (FGF4) secreted by cells and concomitant activation of the MAPK pathway, which drives mESCs to differentiation. Inhibition of the FGF4-mediated differentiation pathway can therefore perpetuate the pluripotent state [16].

Based on this observation, Austin Smith and coworkers pioneered the use of small-molecule inhibitors to block the FGF4 pathway via MEK and GSK3 inhibition, enabling mESCs to grow in minimal, serum-free media. They found that simultaneous inhibition of the MAPK and GSK3 pathways by PD0325901 and CHIRON99021, respectively, allowed robust propagation of mESC cultures with concomitant maintenance of pluripotency (Figure 1(b)) [17]. The pluripotent ground state is achieved by repressing prodifferentiation Mek/Erk/Klf2 axis [18].

Many laboratories started to culture mESCs without serum by using these two small kinase inhibitors, known as 2i. mESCs grown in 2i medium are more homogenous in morphology and exhibit a more uniform gene expression profile than mESCs grown in serum, which now represent a less attractive model system for several reasons. Firstly, serum-cultured mESCs are morphologically heterogeneous, are prone to aneuploidy, and have altered differentiation potential, as a result of fluctuations in the expression of pluripotency and lineage-specific factors. In addition, different serum batches can cause variations between mESC cultures of different laboratories in terms of morphology and gene expression profile due to undefined factor composition [19].

Recent data demonstrated that mESCs do not faithfully mimic ICM cells. It seems that mESCs emerge from a subpopulation of ICM cells that become positive to Blimp1, a germ cell-specific factor, suggesting that mESCs have a germ cell origin and that their derivation is triggered by activation of a transcriptional programme specific to primordial germ cells (PGCs) [20]. All together, these findings demonstrate that mESCs grown in serum are in a metastable condition between ICM cells and epiblast stem cells (EpiSCs), whereas mESCs grown in 2i are in a uniform “ground state” condition, in other words, a condition, which more closely resembles the pluripotent cells of ICM before embryo formation.

Thus, the use of 2i promotes “ground state” pluripotency by blocking factors associated with lineage specification [19] and reducing the fluctuating expression of pluripotency genes observed in serum [21]. In addition, 2i conditions proved to be successful in deriving germline-competent ESCs from all mouse strains and rats [22, 23].

To reveal the true identity of naïve pluripotent stem cells, transcriptome and epigenome comparisons were performed between mESCs grown in 2i and serum.

Genome-wide RNA-Sequencing (RNA-seq) experiments showed that 75% of genes are expressed at similar levels in 2i and serum and pluripotency genes, such as* Oct4*,* Nanog*,* Sox2*,* Rex1*,* Klf2*, and* Klf4*, are similarly expressed. Others, including* c/N-Myc*,* Eras*, and* Id* genes, thought to be essential for serum-grown mESC, are almost absent in 2i-cultured mESCs, suggesting that these genes do not* per se* control pluripotency. Gene-ontology (GO) analysis revealed that genes highly expressed in serum-grown mESCs are enriched in development term, suggesting that mESCs grown in these conditions may be more prone to differentiate. In contrast to this hypothesis, mESCs cultured in serum show similar kinetics and potential to mESCs in 2i medium during* in vitro* differentiation assays. A second term highly enriched in serum-grown mESCs is cell cycle regulation. The doubling time of mESCs is around 10–14 hours, with 65% of cells in S-phase and only 15% in G1-phase. Although data on cell cycle regulation are not conclusive, a recent study showed that G1-phase of mESCs grown in 2i is shorter than that of mESCs cultured in serum, due to increased expression of cell cycle inhibitors [24].

GO analysis of genes highly expressed in 2i revealed a significant enrichment of terms associated with metabolic processes, likely due to inhibition of glucose metabolism by 2i molecules [19]. However, recent efforts showed that other metabolites (i.e., amino acids) control stem cell plasticity, acting as mediators of crosstalk between metabolic flux, cellular signalling, and epigenetic regulation of cell fate [25]. Threonine is the only amino acid required for pluripotency and is intimately associated with S-adenosyl-methionine metabolism (SAM) [26]. Depletion of threonine in mESCs decreases SAM activity with a consequent reduction in trimethylation of lysine 4 on histone 3 (H3K4), resulting in slow growth and increased differentiation.

Recently, other amino acids have been shown to play in this complex scenario. The nonessential amino acid L-Proline acts as a signalling molecule by remodelling methylation profiles of histone 3 on lysine 9 and 36 residues (H3K9 and H3K36), inducing a transition state between mESCs and EpiSCs [27]. The most intriguing feature of mESCs is that they are able to switch their transcriptional profile between 2i and serum conditions, suggesting that the transcriptional state is not stable and that the so-called signature of mESCs only reflects the culture conditions.

Chromatin immunoprecipitation and deep sequencing (ChIP-seq) was used to analyse posttranslational histone modifications: H3K4me3 and H3K36me3 associated with active promoters and transcribed genes; H3K27me3 linked to silencing; H3K9me3 associated with constitutive heterocromatin and imprinted genes. The H3K9me3 ChIP-seq results obtained from mESCs grown in 2i and serum were identical. Genes upregulated in 2i showed increased H3K4me3 marks on active promoters and higher levels of H3K36me3 and a substantial reduction of repressive H3K27me3 mark. In contrast, upregulated genes in mESCs grown in serum showed increased H3K27me3 marks and no significant change in H3K4me3 deposition. Furthermore, the majority of H3K27me3 marks coexisted with H3K4me3 in serum conditions. Extensive studies of these so-called bivalent genes revealed that they are mainly involved in the activation of differentiation and are in paused status. Polymerase II (Pol II) occupancy was found over the coding gene bodies instead of transcriptional start sites [19, 28, 29]. Of these genes, only 33% are conserved in 2i compared to serum, suggesting the existence of a balance between the pluripotency network and lineage specifiers, which inhibit differentiation.

Furthermore, the role of DNA methylation across distinct states was also investigated by mapping genome-wide 5-methylcytosine (5-mC) and 5-hydroxymethylcytosine (5-hmC) in mESCs [30, 31]. While 5-mC represses transcription, elevated 5-hmC levels are associated with increased gene expression. 2i-cultured mESCs display altered distribution of 5-mC and 5-hmC at regulatory elements as well as reduced levels of 5-mC compared to serum. Conversely, EpiSCs show increased 5-mC together with reduced 5-hmC in promoters, in line with their developmental restriction [32].

Switching to 2i induces the rapid onset of “ground-state” gene expression and global DNA demethylation. Mechanistically, a robust hypomethylated state is achieved by repression of* de novo* methylases by PRDM14 and by ten-elevated translocation (TET) 1 and 2-mediated 5-hmC conversion, together controlling transition to “ground-state” pluripotency [32, 33]. Dynamic activation of promoter and enhancers surrounding TET1 and TET2 during the different phases of development controls the progression from pluripotent state toward differentiation [34].

### 2.2. Transcription Factor Heterogeneity in Stem Cells: A Way to Explore the “Future”

The pluripotent state of mESCs is achieved by a coordinated action of gene networks together with multiple signalling pathways responding to environmental cues. This circuitry is established upon formation of pluripotent cells within the blastocyst and persists in epiblast cells until gastrulation [35–37]. At this time, levels of the pluripotency-associated TFs* Oct4* and* Nanog* decrease, and pluripotency can no longer be sustained.

Oct4 was identified over 20 years ago as a transcription factor (TF) specific to early embryogenesis. It is expressed in oocytes, early embryo, embryonic carcinoma cells, and mESCs but is not found in adult differentiated tissues [38, 39]. Expression levels of* Oct4* mRNA is similar in unfertilized oocytes and in zygote, suggesting the existence of maternal transcripts that decrease at the 2- and 4-cell stage of development. Upon zygotic genome activation, upregulation of* Oct4* expression is observed between the morula stage at 2.5 days post coitum (dpc) and the blastocyst stage at 3.5 dpc. Rapid downregulation of Oct4 protein begins in cells differentiating into trophectoderm lineage, while expression levels remain high in ICM cells, in the epiblast at 5.5 dpc, and in germ cell lineage during gastrulation, confirming the role of Oct4 in the maintenance and self-renewal of pluripotent stem cells [40]. No expression is visible from 9.5 dpc onwards, except in PGCs. Thus, Oct4 is spatially and temporally regulated during early murine development and contributes to cell fate decisions. In 7- to 8-day-old mice, Oct4 reaches a minimal threshold level, corresponding to irreversible loss of pluripotency, with concomitant* Nanog* silencing by DNA methylation. This is the point at which the pluripotency network is definitely dismantled [35].

Pluripotency is acquired not only by inhibiting lineage specifiers [41], but also by recruiting chromatin-remodelling complexes to regulatory regions and their binding to closed chromatin domains. Oct4 is therefore emerging as a pioneer TF, able to recruit factors with diverse functions (i.e., TFs, chromatin remodelling proteins) to establish gene-specific programmes [42].

Another intriguing features of pluripotency is its reliance on gene expression heterogeneity, firstly described in a work on the presumptive monoallelic regulation of* Nanog* [43].* Nanog* was originally identified in a genetic screen for molecules that facilitate mESC self-renewal in absence of LIF [44, Chambers, 2007 #300]. Subsequent analyses revealed that* Nanog* acts as a differentiation rheostat through a complex transcriptional and translational regulation able to modify its expression levels without affecting mESC pluripotency [45–47]. It is now known that several TFs are expressed heterogeneously in mESCs depending on the mode of transcriptional as well as posttranscriptional regulation, such as protein synthesis and cell cycle dynamics [48]. TFs heterogeneity is linked to the existence of various functional states in pluripotent stem cells and to the fact that individual cells in a mixed population have a propensity either to self-renew or commit to differentiation. Thus, the working hypothesis is that stochastic fluctuations of TFs provide opportunities for mESCs either to modulate their potential fate or to remain pluripotent by exploring multiple lineage options. This mechanism confers robustness to such an important cell population as pluripotent stem cells by preserving both the identity of cells and their capacity to differentiate in response to different signalling pathways.

### 2.3. Human Embryonic Stem Cells

The first human embryonic stem cells (hESCs) were derived from blastocysts produced by* in vitro* fertilization (IVF) in 1998 [5], using immunosurgical procedures similar to those utilized by Evans and Kaufman 17 years earlier to derive the first mESC line. In the following years, few other hESC lines were obtained [49, 50] until 2004, when a new standardized protocol and a well-defined culture medium allowed efficient derivation of hESCs [51].

The establishment of hESC lines showed that, like mESCs, hESCs can grow indefinitely* in vitro*, maintaining their karyotype and pluripotent capacity [5, 49–51]. The undifferentiated state of hESCs and their developmental potential to differentiate into all cell types of the body were confirmed by several assays* in vitro* and* in vivo* [52].

The most commonly used* in vivo* method for determining whether a hESC line is pluripotent is transplantation in immune-deficient mice to assess its ability to form teratomas. A teratoma is a nonmalignant tumour composed of a disorganized mixture of cells and tissues deriving from the three germ layers [53]. Formation of primate chimeras, the most stringent method to test pluripotency in rodents, cannot be undertaken in human, due to ethical issues. To overcome this barrier, interspecies chimeras have been generated through injection of hESCs into mouse blastocysts [54]. Although it has been shown that hESCs can be integrated into the ICM, human cells poorly contribute to mouse embryo suggesting that the method is inefficient [54]. Therefore, we still lack a definitive functional assay to test pluripotency in human.

### 2.4. Differences between Mouse and Human Embryonic Stem Cells

**Figure 2:**
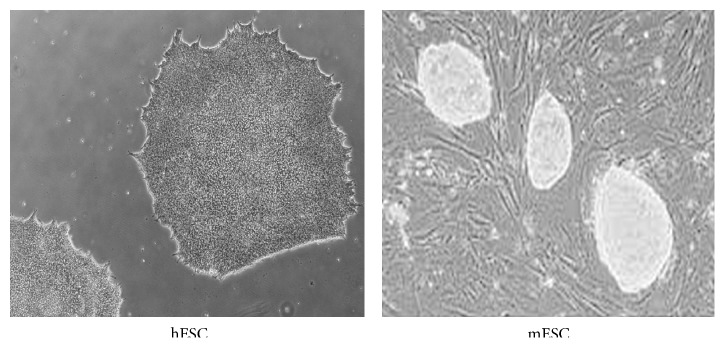
Morphology of hESCs and mESCs.

mESCs and hESCs show equivalent developmental potential, as would be expected considering their common embryonic origin. However, several reports have described significant differences between the two cell lines. In terms of colony morphology, hESCs grow as flat and compact colonies unlike the multilayered and rounded colonies formed by mESCs [5] (Figure 2). Other important differences concern growth conditions, transcriptional networks, signalling pathways that controls self-renewal and pluripotent state, and epigenetic signatures [55, 56].

To decipher the transcriptional network in hESCs, it is crucial to understand the mechanism(s) regulating pluripotency. Several studies have shown that maintenance of self-renewal and pluripotency in hESCs involves very different signalling pathways from those in mESCs. While mESCs are dependent on LIF and Bmp4, LIF is dispensable for hESCs [57, 58], and the presence of BMP4 in culture medium induces hESC differentiation into trophoblasts [59]. hESCs also require other factors such as Activin A/Nodal, FGF2 [60–62] and IGF [63]. Activin A and Nodal, members of the transforming growth factor (TGF-*β*) superfamily, play a role in maintaining hESC self-renewal by promoting NANOG transcription via SMAD2/3 signalling [60–62]. SMAD2/3 proteins directly bind and regulate expression of NANOG [64]. In addition, recent ChIP-seq analysis of hESCs showed the binding of SMAD2/3 to promoter regions of other genes involved in self-renewal such as OCT4, TERT, MYC, and DPPA4, with SMAD2/3 sharing approximately one-third overlap with NANOG genomic targets, suggesting their cooperation in transcriptional control of pluripotency genes [65].

Another report showed that SMAD3 cooccupies OCT4 genomic binding sites across the genome in both hESCs and mESCs [66], indicating a more extensive involvement of SMAD2/3 signalling in sustaining pluripotency in hESCs.

Unlike Nodal and Activin A, the exact mechanism(s) by which FGF2 signalling is able to sustain hESC pluripotency is still not clear. This is partly due to the complexity of pathways influenced by FGF signalling and to the varying culture conditions employed by different laboratories. FGF2 signalling was reported to sustain NANOG expression by cooperating with Nodal and Activin A signalling [61, 67] through the MEK/ERK pathway [68]. However, the way in which the MEK/ERK pathway regulates NANOG expression remains essentially unknown. Large-scale analyses undertaken to profile the global phosphoproteome in hESCs after FGF2 stimulation identify phosphorylated players of canonical pathways involved in self-renewal and pluripotency, such as PI3K/Akt, MAPK/ERK, and Wnt, as well as pluripotency regulators including OCT4, SOX2, RIF1, SALL4, DPPA4, and p53 [69, 70]. These data suggest possible events occurring downstream of FGF2-FGF receptor interaction. In addition to sustaining expression of pluripotency-associated genes, the FGF and Activin A/Nodal pathways synergize to inhibit BMP signalling [64], which represses self-renewal and promotes differentiation by SMAD1/5/8 binding to NANOG promoter, thereby inhibiting its expression.

IGF has also been implicated in pluripotency maintenance since it was observed that blocking the IGF2-IGF1 receptor pathway reduced survival and clonogenicity of hESCs [63]. IGF is able to sustain pluripotency through activation of the PI3K pathway. Inhibition of either IGF or PI3K signalling efficiently promotes differentiation of hESCs [71]. PI3K maintains hESC pluripotency by suppressing Activin A/Nodal-triggered pathways [71], thus redirecting SMAD2/3 activity to pluripotency rather than differentiation. In conclusion, PI3K/Akt cooperate with Activin A to promote a pluripotent state and that Activin A has context-dependent functions in promoting and antagonizing self-renewal pathways.

OCT4, NANOG, and SOX2 form the pluripotency-regulating network in hESCs, as in mESCs [72]. RNA interference-mediated knockdown of these genes in hESCs results in loss of pluripotency and self-renewal [73–75].

ChIP technologies were used to map the genomic binding sites of these proteins in hESCs [76, 77]. Extensive OCT4, NANOG, and SOX2 cobinding was found at numerous genomic target sites localized in active as well as in silent genes, supporting their role in pluripotency and self-renewal through autoregulatory activation, and repression of key genes involved in developmental processes [77].

Although mESCs and hESCs share the same pluripotent transcriptional circuitry, a limited overlap was observed between OCT4 and NANOG target genes, suggesting differences in the networks controlled by the two TFs in the two species [76, 77]. This result was confirmed by other techniques including microarray, serial analysis of gene expression (SAGE), and massively parallel signature sequencing (MPSS) [78, 79].

Other epigenetic signatures of hESCs, different from their mouse counterparts, are exemplified by X chromosome status. Female mESCs are in a pre-X inactivation state carrying two active X chromosomes (X_a_X_a_). Upon differentiation, one of the two X chromosomes becomes transcriptionally silenced (X_i_X_a_) through the X-chromosome inactivation (XCI) process [80]. The active X chromosome therefore represents an epigenetic hallmark of an undifferentiated state in female mESCs. By contrast, XCI is already established in the majority of undifferentiated female hESCs. XCI status was reported to vary greatly between different hESC lines and subcultures of a single cell line [81–83]. Three distinct states of XCI were described for hESCs [84, 85]. Class I cells possess two active X chromosomes (X_a_X_a_) and, like mESCs, X-inactive specific transcript (XIST) is upregulated, coats X chromosome, and triggers accumulation of H3K27me3, upon differentiation. However, hESCs in the pre X-inactivation state* in vitro* are epigenetically unstable, readily proceeding toward class II and subsequently class III [86, 87]. Class II cells contain one inactivated X chromosomes (X_a_X_i_), coated by XIST and marked by H3K27me3. Class II hESCs may further progress toward class III [87], where the silent state of the inactive X chromosome is largely maintained, while XIST expression and the H3K27me3 histone mark are lost, leading to partial reactivation of some X_i_-linked genes [88]. In class III cells, XIST is silenced through methylation of its promoter, and upon differentiation class III cells do not reexpress XIST and do not acquire H3K27me3 marks [88].

A recent publication reported that oxygen tension is one factor favouring the establishment of XCI in hESCs. Derivation and maintenance of hESCs are conventionally performed in atmospheric concentration (20% O_2_) [5, 49–51], which is a hyperoxic condition compared to physiological levels (5% O_2_) [89]. hESCs derived at 5% O_2_ tension preferentially remain in class I, as demonstrated by the absence of XIST expression, high levels of methylation of XIST promoter, biallelic expression of X-linked genes, and the ability to undergo random X inactivation upon differentiation [88]. When cells derived and grown in hypoxia conditions are exposed and cultured at 5% O_2_, they show signs of XCI progressing irreversibly toward class II and then toward class III [88]. This finding strongly supports the observation that hESCs are epigenetically unstable* in vitro* in terms of XCI, since it was demonstrated that human preimplantation blastocysts contain cells in the pre-X inactivation state [90].

A successful conversion of XIST-dependent class II cells into class I was reported in another work, in which hESC medium was supplemented with sodium butyrate, a histone deacetylase inhibitor (HDACi), and an S-adenosylhomocysteine (SAH) hydrolase inhibitor (DZNep), able to deplete cellular levels of enhancer of zeste homolog 2 (EZH2) and to remove H3K27me3 marks from the genome [91, 92].

Derivation in a naïve state was reported to be unsuccessful for class III hESCs [91]. In addition, class I hESCs were derived in normoxic conditions by adding both sodium butyrate and DZNep to culture medium, suggesting that these two molecules are able to prevent XCI and maintain the cells in class I for several passages [92].

To date, there are no reports in the literature of any other epigenetic signature characterizing hESCs, and it would be interesting to discover whether the instability associated with the inactivation process reflects a more general epigenetic instability in hESCs.

### 2.5. Defining the Pluripotent “Ground State” in hESCs

Differences in morphology, growth factor dependency, and epigenetic modifications observed in mESCs and hESCs were initially thought to reflect species-specific variations of pluripotency. This idea was challenged when EpiSCs were isolated from epiblasts of postimplantation murine blastocysts [93, 94]. Although EpiSCs fulfilled some crucial criteria of pluripotency (i.e., teratoma formation, expression of pluripotency-associated TFs), transcriptome analysis showed that independent EpiSC lines were similar to each other, but different from mESCs. Interestingly the gene expression profile of EpiSCs is more similar to that of postimplantation epiblast than preimplantation ICM, consistent with their embryonic developmental stage [93, 94]. The observed differences between mESCs and EpiSCs indicate the existence of two distinct pluripotent states, recently termed* naïve* and* primed*, respectively, belonging to two different developmental stages [95].

**Figure 3:**
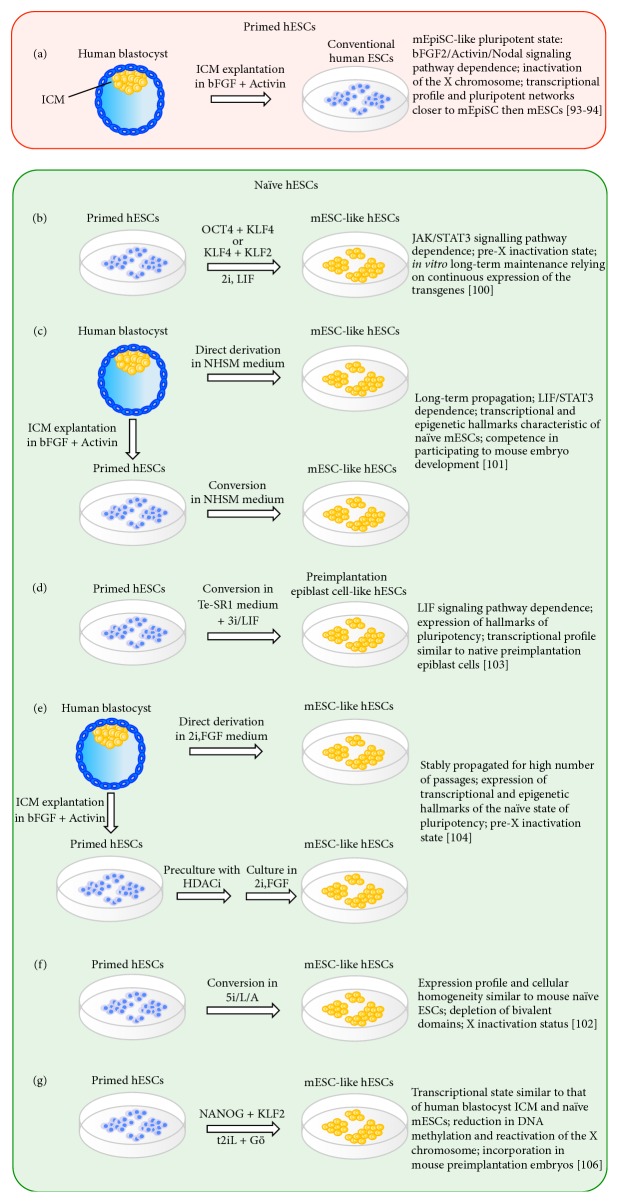
Schematic representation of culture conditions used to obtain primed and naïve pluripotent hESCs. (a) Conventional hESCs are derived from human blastocysts using a culture medium supplemented with bFGF and Activin A. hESCs derived in conventional culture conditions exhibit a pluripotent state more similar to murine EpiSCs than ESCs and are defined as primed hESCs. (b) hESCs with mESC-like characteristics can be produced by induced expression of either OCT4/KLF4 or KLF4/KLF2 transgenes in primed hESCs. Stable* in vitro* maintenance of these naïve hESCs requires continuous expression of the transgenes. (c, d, and e) Defined conditions allowing derivation of naïve hESCs from either already established primed hESCs or directly from blastocysts without the use of pluripotency-associated transgenes. (f) Conversion of primed hESCs into naïve state using five kinase inhibitors (5i) + LIF + Activin. (g) Short term expression of NANOG and KLF2 in primed hESC cells is sufficient to trigger ground state in hESCs cultivated in 2iL medium plus PKC inhibitor Gö6983.

Interestingly, hESCs share many features with EpiSCs (Figure 3(a)). Like hESCs, EpiSCs grow as flat and compacted colonies and show intolerance to passaging at single cells, and their derivation and long-term maintenance are strictly depended on FGF2 and Activin, but not on LIF and/or BMP4 as is the case with mESCs [93, 94, 96]. Again, like hESCs, EpiSCs are able to differentiate into trophectoderm in presence of BMP4 [59, 97], whereas mESCs have little or no ability to contribute to trophectoderm lineages in chimeric embryos [98]. ChIP experiments in mESCs, EpiSCs, and hESCs to identify OCT4 target sites showed a limited overlap of OCT4 targets in hESCs and mESCs [76, 77], but a 7-fold greater overlap between hESCs and mEpiSCs [94], suggesting that similar transcriptional networks are able to maintain pluripotency in both hESCs and EpiSCs. In addition, female EpiSCs resemble female class II hESCs by the presence of an inactive X chromosome coated by Xist RNA and enriched for H3K27me3 marks [99].

One plausible scenario is that, during derivation and propagation, hESCs are unstable in conventional culture conditions and progress toward an EpiSC-like pluripotent state, and they are thus called primed hESCs [95] (Figure 3(a)).

The question was, did the naïve state exist for hESCs? In 2010, Rudolf Jaenish and coworkers demonstrated that it is possible to revert primed hESCs into a naïve state by ectopic expression of OCT4 and KLF4 or KLF4 and KLF2 plus 2i/LIF medium [100] (Figure 3(b)). These naïve hESCs appeared almost completely morphologically indistinguishable from mESCs, growing as packed dome colonies. In addition, maintenance of self-renewal depended on the JAK/STAT3 pathway, in contrast to conventional hESCs, which require Activin A signalling. Furthermore, the conversion of female primed hESCs into naïve hESCs is accompanied by X chromosome reactivation and changes in methylation of XIST promoter region, consistent with pre-X inactivation state [100]. These data supported the* bona fide* conversion of hESCs into a more immature state, although long-term propagation of naïve hESCs required constitutive expression of Klf4/Oct4 or KLf4/Klf2 transgenes. The use of forskolin led to transient induction of* KLF4* and* KLF2* expression. These genetically unmodified forskolin-treated naïve hESCs could not be maintained for more than 15–20 passages, at which point they stopped proliferating and differentiated [100].

In 2013, the laboratory of Hanna established an optimized chemically defined medium, termed naïve human stem cell medium (NHSM), allowing robust and long-term maintenance of naïve hESCs [101]. These growth conditions were used to obtain naïve hESCs both from reversion of primed hESCs and from preimplantation blastocysts (Figure 3(c)). Transcriptional and epigenetic analyses revealed a functional overlap between mESCs and naïve hESCs. In addition, Gafni and coworkers showed that GFP-labelled hESCs microinjected into murine morulae at 2.5 dpc colonized different tissues of chimeric murine embryo, indicating a functional competence [101]. Interestingly, the naïve cells showed a higher integration into the ICM compared to that of primed pluripotent cells, suggesting that the generation of interspecies chimeras, through hESC injection into murine morulae, might be used as stringent assay to test human naïve pluripotent state. However, the method does not seem to be reproducible to be used as a routine functional assay and further improvements are necessary [102].

The combination of three small molecules, the PI3K inhibitor PD0325901, the GSK3 inhibitor BIO, and the BMP signalling inhibitor Dorsomorphin, with LIF (referred to as 3iL) allowed conversion of hESCs into a naïve-like state (Figure 3(d)). hESCs cultured in 3iL showed LIF signalling-dependence and hallmarks of pluripotency including elevated expression of NANOG, KLF4, DPPA3, and TBX3 [103].

Transcriptome analysis confirmed the naïve state of hESCs cultured in 3iL. In addition, the different expression profile of 3iL hESCs compared to conventional hESCs is accompanied by global histone modification changes, resulting in derepression of preimplantation epiblast genes, as well as changes in OCT4, NANOG, and p300 binding sites, suggesting a rewiring of the pluripotency network [103]. The 3iL-induced hESC state narrows the gap between* in vivo* and* in vitro* pluripotent states.

Conversion of primed hESCs into naïve cells was also obtained by preculturing cells in HDACi, such as sodium butyrate and suberoylanilide hydroxamic acid (also known as SAHA or Vorinostat), followed by culture in 2i medium supplemented with FGF2 (2iF) [104] (Figure 3(e)). Preculturing in HDACi is an essential requisite for conversion into a naïve state, since exposure of primed hESCs to 2i induces differentiation. FGF2 is also a necessary component [104], supporting previously published data [101]. Using 2iF, it was also possible to derive naïve cells directly from preimplantation human embryo. Curiously, when naïve hESCs directly derived from an embryo in 2iF were switched to 3iL or to 2i, cells were able to grow stably for more than 60 passages, unlike cells grown in 2iF alone; though an increase in differentiation compared to 2iF conditions was observed [104].

Recently, Rudolf Jaenisch and coworkers have identified a combination of five kinase inhibitors (5i) able to induce and maintain in conventional hESCs* OCT4* distal enhancer activity [102], an established molecular signature of “ground-state” pluripotency [94, 105]. Indeed, provision of 5i supplemented with LIF and Activin A (5i/L/A) enables both conversion of primed into naïve hESCs in absence of reprogramming factors and the direct derivation of naïve ES cells from human blastocysts [102] (Figure 3(f)). The authors compared their optimized culture conditions with those previously reported to induce a naïve pluripotent state [101, 103, 104]. Remarkably, substantial differences have been observed among the different cell lines in terms of* OCT4* distal enhancer activity and transcriptional profile of markers typically associated with the self-renewal and pluripotency of mESCs.

Immediately after the publication of the Jaenish laboratory, Austin Smith and coworkers have described the production of naïve hESCs by short-term expression of NANOG and KLF2 transgenes in primed cells. The rewired cells culturing in 2iL medium in combination with the protein kinase C (PKC) inhibitor Gö6983 (t2iL + Gö) has been shown to sustain the pluripotent “ground-state” in absence of transgene expression [106] (Figure 3(g)). Interestingly the authors compared the transcriptional state of t2iL + Gö cells, conventional human PSCs, human blastocyst ICM, and naïve mESCs with NHSM, 3iL, and 5i/L/A cells reported to have undergone conversion to a naïve state [101–103]. The authors observed that while the transcriptional profile of t2iL + Gö cells most resembled that of human blastocyst ICM and naïve mES cells, a significant difference from the conventional state was not apparent for lines cultured in NHSM, 3iL [106]. Upregulation of naïve markers and downregulation of lineage markers appeared comparable between t2iL + Gö and 5i/L/A hESCs; however some differences could be observed in the expression of epigenetic regulators, such as DNMT3A and TET1. In addition, Jaenish and coworkers reported X chromosome inactivation in contrast to t2iL + Gö hESCs.

All those findings suggest that, similarly to mouse, a naïve “ground-state” of pluripotency exists for hESCs. However, the initial comparative studies of the naïve human cell lines so far produced clearly indicate that the various culture conditions induce different pluripotent states, each showing similar features, but not identical, to those of naïve mESCs. The question whether differences exist in the pluripotent “ground-state” between human and mouse is still not clear. In addition, the off-targets effects of inducers/inhibitors added to culture medium should be taken in account: heterogeneity increases when using a broader combination of different inducers/inhibitors.

Further comparative studies of mouse and human preimplantation development and more extensive comparisons of the different naïve hPSCs are required to find an answer.

## 3. Induced Pluripotent Stem Cells

### 3.1. Cellular Reprogramming

The first evidence that somatic cells can be reprogrammed into a pluripotent state came from somatic cell nuclear transfer (SCNT) experiments performed in 1962, in which the nucleus of a differentiated cell was introduced into an enucleated oocyte giving rise to a cell, which, after stimulation, was capable of developing into an organism [111].

Four decades later, the possibility to revert the potency state of somatic nuclei was confirmed by fusion with ESCs [112, 113], suggesting that both unfertilized eggs and ESCs contain factors that are able to reprogramme somatic cells. Subsequently, in 2006 Takahashi and Yamanaka identified four genes, Oct4, Sox2, Klf4, and cMyc (OSKM) [6], which, when simultaneously overexpressed, are sufficient to induce reprogramming of mouse skin fibroblasts into pluripotent cells and called them induced pluripotent stem cells (iPSCs). Using the OSKM cocktail, iPSCs were also generated using human fibroblasts [7].

Many other groups have since reported reprogramming of several murine and human cell types, demonstrating the simplicity and reproducibility of the methodology, which can be applied to reprogramme most, if not all, somatic cells.

However, the efficiency of converting somatic cells into iPSCs is dramatically low; only approximately less than 1% of transfected fibroblasts become pluripotent. Extensive studies have been performed, modifying the OSKM cocktail in an attempt to improve efficiency. An exhaustive overview of those studies has been recently reported elsewhere and is therefore not further discussed here [114].

### 3.2. The Pluripotent “Ground State” of iPSCS

Murine iPSCs share all features of naïve mESCs, including morphology, expression of pluripotency-associated TFs, reactivation of X chromosome, ability to form teratomas, contribution to the germline of chimeric mice obtained by blastocyst injection, and generation of mice by tetraploid complementation [115–118].

**Figure 4:**
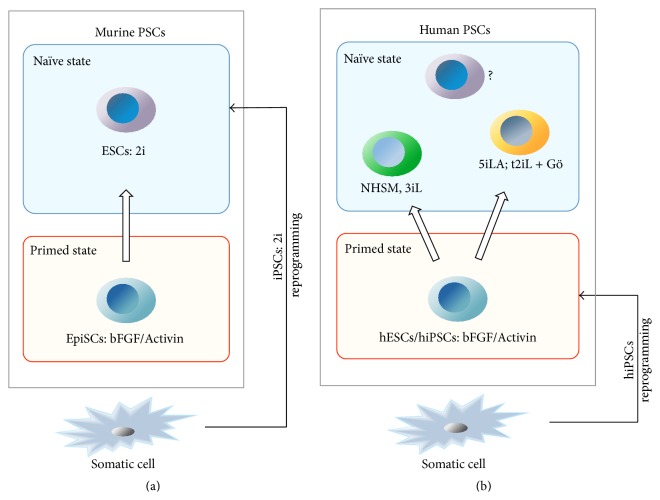
Visual comparison of murine and human PSCs. (a) In mouse, ESCs and EpiSCs are characterized by a different pluripotent state, called naïve and primed, respectively, which reflects their embryonic origin. The stability and homogeneity of mESCs cultured in 2i represent a developmental ground state closely reflective that of the ICM of preimplantation blastocysts. Pluripotent stem cell can be obtained also by reprogramming of somatic cells. Mouse iPSCs, cultured in 2i, show a ground state similar to mESCs. (b) Human ESCs and iPSCs cultured in presence of bFGF/Activin are in the primed state. Different conditions have been established to convert primed hESCs and hiPSCs into a naïve state. The initial comparative analysis of the naïve human cell lines clearly indicates that the various culture conditions induce different pluripotent states, each showing similar features, but not identical, to those of naïve mESCs. Whether the authentic pluripotent ground state of naïve hESCs is identical to that of mESCs, it is still to be determined.

Similarly to mouse, human iPSCs express hESC-specific surface antigens, including stage-specific embryonic antigen- (SSEA-) 3 and 4, tumour-related antigens (TRA-1-60, TRA-1-81), pluripotency-associated TFs, high telomerase activity, and the ability to differentiate into cells of the three germ layers by teratoma formation [7, 119–121]. However, human iPSCs are in a primed state, as suggested by the presence of inactive X chromosome in female iPSCs [122–125]. As with hESCs, conversion of human iPSCs into a naïve state can be achieved by culturing in defined media, as previously discussed. A visual comparison of murine and human PSC is shown in Figure 4.

### 3.3. Barriers to the Reprogramming Process

Although cell reprogramming is reproducible, the process is slow (around 2 weeks) and inefficient. Only a small fraction of transfected cells (0.1–3%) become iPSCs [126], indicating that somatic cells must overcome barriers to revert to pluripotent state.

**Figure 5:**
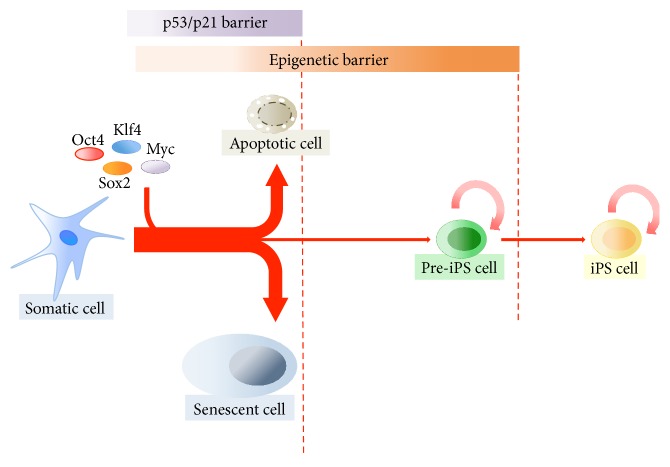
Scheme depicting cellular reprogramming and barriers affecting efficiency of the process.

Apoptosis and senescence are the ultimate fate of the majority of cells induced by OSKM. Various reports showed that expression of Yamanaka factors in murine and human fibroblasts is able to induce p53 and p21^CIP1^ [127–129]. Knockdown of p53 and/or induction of reprogramming in p53-null MEFs increase the efficiency of iPSC colony formation [127, 129, 130], suggesting that the p53/p21 pathways represent a barrier to reprogramming (Figure 5). Several groups also observed that expression of reprogramming factors activates the DNA damage response (DDR) pathway [128–130]. Thus, p53 might prevent the reprogramming of DNA-damaged cells by inducing apoptosis and senescence. Activation of the p53/p21 pathways highlights the tremendous stress to which cells are subjected during the process of reprogramming. Although reprogramming efficiency can be improved by interfering with crucial antiproliferative genes, the drawback of blocking important pathways that protect the cell from detrimental mutations is that it might affect the safety of resulting iPSCs, especially for medical applications.

The acquisition of pluripotency during reprogramming is accompanied by epigenetic remodelling of somatic cells, necessary to establish the transcriptional and epigenetic landscape defining the pluripotent state. Somatic cell identity is maintained and stabilized by epigenetic mechanisms, such as DNA methylation and histone modifications, which represent a potent barrier to the reprogramming process [126]. Reports by different groups showed that, in both mouse and human, iPSCs may retain residual transcriptional and epigenetic signatures characterizing their somatic origin [131–136]. Incomplete erasure of tissue-specific DNA methylation and aberrant* de novo* methylation during reprogramming partially explain the persistent expression of somatic genes in iPSCs [133, 135]. Inhibition of DNA methylation by 5-azacytidine (5′-AZA) or downregulation of DNA methyltransferase 1 (Dnmt1) increases reprogramming efficiency, demonstrating the functional linkage between DNA methylation and reprogramming [137].

All together, these findings provide evidence that DNA methylation is an obstacle to iPSC reprogramming. Trimethylation at H3K9me3, associated with constitutive heterochromatin and imprinted genes, is also a barrier in somatic cell reprogramming [42, 138, 139]. Several studies reported a functional linkage between H3K9me3 and reprogramming, showing that downregulation of either histone methyltransferases (HMTs), such as Setdb1, Ehmt1, Ehmt2, Suv39H1, and Suv39H2, or heterochromatic protein 1-*γ*, an H3K9me3 reader, enhances reprogramming efficiency.

iPSC reprogramming is a dynamic process involving several steps leading to repression of the somatic gene programme and reexpression of pluripotency-associated TFs. In pre-iPSCs, a clonal population of cells having already acquired an ESC-like morphology, the somatic gene expression programme is repressed, but pluripotency-associated TFs are not yet expressed, and they thus represent partially reprogrammed cells. Chromatin mark analysis of pre-iPSCs and MEFs showed enrichment of repressive H3K9me3 marks compared to iPSCs stimulated by BMP signalling [137]. BMP effectors, such as Smad proteins, have thus been proposed as interacting with H3K9 HMTs, including Setdb1 and Suv39H1, to promote H3K9 trimethylation. Accordingly, knockdown of HMTs, such as Setdb1, Ehmt1, and Ehmt2, or of HP1*γ* in pre-iPSCs induces upregulation of the pluripotency genes Nanog, Gdf3, Zfp42, Dppa4, and Lin28 [138]. These findings suggest that erasure of the H3K9me3 histone mark is a necessary step for reprogramming into naïve iPSCs.

A study mapping the initial interaction of OSKM factors with the human genome during the first 48 hours of reprogramming revealed the existence of genomic regions inaccessible to OSKM binding and enriched for H3K9me3 marks in pluripotency genes, such as Nanog and Sox2 [42]. These regions are refractory to OSKM binding due to the presence of H3K9me3 marks. Knockdown of SUV39H1/H2 during human iPSC reprogramming facilitates the access of OSKM factors to these regions, enhancing the efficiency and kinetics of reprogramming [42]. In a previous study, Lister and colleagues reported the identification of large regions showing aberrant non-CpG island methylation in human iPSCs not observed in ESCs [134]. These methylation “hot spots” enriched for H3K9me3 marks perfectly overlap with the OSKM binding regions identified by Soufi et al. [42, 140], supporting the hypothesis that heterochromatic marks disrupt the correct DNA methylation patterns in these regions and contribute to the “epigenetic memory” of iPSCs.

### 3.4. Advances in iPSC Technology: Toward Reprogramming Using Small Molecules

The discovery that somatic cells can be converted into a pluripotent state opened up the possibility of using iPSCs in cell transplantation therapies, as discussed later in this review. However, iPSC generation still presents several drawbacks, limiting the use of this promising resource in clinical applications.

The first iPSC reprogramming method utilized retroviral or lentiviral vectors for expression of OSKM factors. Although these vectors ensure high reprogramming efficiency, they can cause insertional mutagenesis resulting in harmful effects, such as tumour formation. Thus, for cell therapy purposes, alternative vectors were used to generate transgene integration-free iPSCs, including adenovirus [141], piggyBac transposon [142], episomal vectors [143], Sendai virus [144], plasmids [145], minicircle vectors [146], proteins [147], and synthetic RNAs [148]. However, achieving full reprogramming by reactivation of key pluripotency markers is a more lengthy process.

Another important issue that needs to be addressed is the presence in the cocktail of c-Myc, which may increase the risk of tumour formation. Mice generated by either blastocyst injection or tetraploid complementation with iPSCs are prone to develop tumours [149–151].

Current research in reprogramming is moving toward the development of transgene-free methodologies based on small molecules. Different compounds promoting dedifferentiation by acting on signalling pathways and epigenetic mechanisms have been identified.

The use of 5′-AZA and 2i was found to facilitate the transition of partially reprogrammed MEFs to fully reprogrammed iPSCs by promoting DNA demethylation [137, 152, 153].

HDACi, such as valproic acid, SAHA, and trichostatin A were shown to increase efficiency in recovering iPSC colonies [154, 155]. Valproic acid proved to be the most potent in enabling efficient reprogramming of murine fibroblasts in absence of c-Myc [154] and human fibroblasts in absence of Klf4 and c-MYC [155]. Sodium butyrate was also reported to increase dedifferentiation of human fibroblasts in absence of both Klf4 and c-MYC transgenes [156].

Kenpaullone (KP), a GSK3*β* and CDK/cyclin complexes inhibitor (CDK1/cyclinB, CDK2/cyclinA/E, and CDK5/p25), was identified by molecule library screening as being able to replace Klf4 factor and increase reprogramming efficiency via an as yet unclear mechanism [157].

It has shown that the inhibition of TGF-*β* signaling increases induction of iPSCs, replacing the requirement of Sox2 and c-Myc [158]. Accordingly, a high-content chemical screening of small molecules led to the identification of the Tgf*β*R1 inhibitor, E-616452, also called RepSox, able to induce reprogramming of MEF cells in absence of both Sox2 and c-Myc [159]. Although RepSox is able to replace Sox2 during reprogramming, it would act by inducing* Nanog* rather than* Sox2* expression [159]. Other molecules have been shown to functionally replace Sox2, including inhibitors of the Src family kinases [160], CHIR99021 [161], and a combination of a L-channel calcium agonist, BayK8664 (BayK), with the G9a histone methyltransferase inhibitor BIX01294 [162].

Vitamin C enhances the efficiency of iPSC reprogramming using both mouse and human fibroblasts [163], by inducing demethylation of H3K36me2/3 marks, mediated by Jumonji domain-containing histone demethylases (Jhdm) 1a and 1b [164]. In combination with vitamin C, forced expression of Jhdm1a was able to replace Klf4 and c-Myc, while Jhdm1b was able to replace Sox2, Klf4, and c-Myc confirming the key role of these enzymes in reprogramming. Furthermore, Jhdm1b represses the Ink4/ARF locus, a known block in reprogramming involved in senescence [165], by reducing H3K36me2/3 marks. In addition, Jhdm1b might cooperate with OCT4 for activation of the miR-302/367 miRNA cluster, probably facilitating OCT4 access [164].

In addition, generation of iPSCs via OSKM results in aberrant methylation of the imprinted Dlk1-Dio3 gene cluster, associated with the inability or reduced capacity of iPSCs to generate mice via tetraploid blastocyst injections, suggesting that stable repression of this locus is another roadblock in the reprogramming process [166, 167]. Vitamin C improves iPSC generation by counteracting epigenetic silencing of the Dlk1-Dio3 locus. Vitamin C was shown to preserve an active histone configuration at Dlk1-Dio3 locus by preventing recruitment of Dnmt3a and consequent hypermethylation of the region, leading to stable silencing of maternal Dlk1-Dio3 transcripts [166].

Interestingly, a recent report showed that addition of vitamin C to mESCs promotes TET activity, leading to an increase in 5-hmC, followed by DNA demethylation of crucial gene promoters involved in pluripotency, suggesting another mechanism by which vitamin C might act during reprogramming [168].

Li and coworkers have identified a combination of small molecules, including VPA, CHIR99021, E-616452, and tranylcypromine (VC6T), able to induce MEFs reprogramming in combination with a single transgene, Oct4, thus replacing Sox2, Klf4, and c-Myc [169]. It has been proposed that VC6T may facilitate miPSCs generation by lowering several barriers, such as epigenetic modifications and intracellular signaling pathways, during reprogramming [169]. Similarly, Yuan and coworkers have identified a different combination of small molecules, including AMI-5, a protein arginine methyltransferase inhibitor, and A83-01, a TGF-*β* inhibitor, that enable the production of miPSCs from MEFs using only* Oct4* transgene expression [170].

Currently, in mouse it is possible to substitute the OSKM factors completely, avoiding the risk of insertional mutagenesis and with resulting iPSCs fulfilling all pluripotency and differentiation criteria. Hou and coworkers have shown a combination of small molecules, including VC6T, forskolin, and DNAZep (VC6TFZ), that enable MEFs reprogramming into pluripotent cells at a frequency of 0.2% [171]. Furthermore, the authors identified in forskolin, CHIR99021, E-616452 and DZNep (C6FZ), critical and sufficient molecules to induce iPSCs reprogramming from MEFs in absence of OSKM, although with 10-fold lower efficiency compared to VC6TFZ [171]. This study clearly shows that somatic reprogramming can be achieved using small molecule compounds. However, that goal has not been achieved yet in human.* Oct4* transduction in combination with chemically defined compounds is necessary to generate efficiently hiPSCs [172–174], suggesting the needing to improve in human the reprogramming technology in perspective of cell-based therapies.

## 4. Pluripotent Stem Cells: Applications, Problems, and Future Directions

Self-renewal and pluripotency are fundamental characteristics of ESCs and iPSCs, making them attractive to academia and industry for their potential preclinical and clinical applications in the treatment of a wide array of diseases and pathological conditions.

Regenerative medicine is an exciting and fast moving field of research with the ambitious aim of using stem cells to replace tissues/organs damaged by injury, disease, or congenital defects. Because of their ability to differentiate into all the specialized cell types of an adult, pluripotent stem cells (PSCs), including human ESCs and iPSCs, are a promising source for cell-based therapies [175]. Therapeutic potential of PSCs has been evaluated in preclinical studies, where PSCs transplantation has been applied to treat different diseases [176–185]. Noteworthy, cell therapy into animal models has shown beneficial effects, such as restoration of locomotion after spinal cord injury with hESC-derived oligodendrocyte [177], improved vision with hESC-derived retinal pigment epithelium (RPE) in blindness models [181], and improved cardiac function in a porcine ischemic cardiomyopathy model with iPSC-derived cardiomyocytes [186].

The potential benefit of both human ESCs and iPSCs has been also assessed in human clinical trials, summarized in Table 1. Early benefits to patients have been reported in some of these on-going trials, although final reports are few or not easily accessible [107].

In 2010, Geron Corporation launched the first clinical trial to evaluate the safety of hESC-derived oligodendrocyte progenitor cells (OPCs) in the treatment of spinal cord injuries (Table 1). Asterias Biotherapeutic continued this study, when Geron Corporation discontinued the cell therapy program. Although no official publication has been released, the results from the clinical trial have been presented at the American Society for Gene and Cell Therapy (ASGCT) in 2014. The data showed no serious adverse events due to the transplantation and, in four out of five patients, Magnetic Resonance Imaging (MRI) scan showed some positive effects in reducing the deterioration of spinal cord tissue.

In 2011 Ocata Therapeutics started two prospective clinical studies to establish safety and tolerability of subretinal transplantation of hESC-derived retinal pigment epithelium (RPE) in patients with Stargardt's macular dystrophy and dry age-related macular degeneration (AMD), two leading cause of blindness in the developed world [187, 188]. The results of these studies provided evidence of medium-term safety, graft survival, and possible biological activity of pluripotent stem cell progeny in individuals affected by a disease [188]. Chabiotech Ltd. launched a phase I/IIa study to evaluate safety and tolerability of subretinal transplantation of hESC-derived RPE cells in two patients with advanced dry age-related macular degeneration (AMD) and two with Stargardt's macular dystrophy. Preliminary results showed no adverse events (tumour growth or other unexpected effects) and safety related to the therapy. Visual activity has been found to be improved in three out of four treated patients [189].

However, quality and safety issues must be fulfilled to produce clinical-grade human iPSCs suitable for cell therapies [187] and different issues still need to be addressed before translation of human iPSCs in clinical practice.

The use of viral systems to deliver reprogramming factors, which leads to permanent integration of oncogenes and results in potentially harmful genomic alterations, is a serious concern [188]. To overcome those issues, in mouse, a protocol transgene-independent that uses small molecules has been recently established, although the strategy is less efficient than viral transduction with OSKM factors and the reprogramming process is a more lengthy process [171]. In human, the same goal has not achieved yet and* Oct4* transduction is required [172–174], suggesting the need to optimize the reprogramming strategies for the clinical safety of hiPSCs and improve the efficiency of the process.

Recent studies have provided evidence of genetic and epigenetic variations between different iPSC lines [189]. Some of the variations may be inherited from donor somatic cells or acquired during either the reprogramming process or extensive culturing [189]. Although such genetic and epigenetic variation affect only a small portion of the genome, they may change the properties of iPSCs and their derivatives, resulting in increased risk of tumorigenicity, altered differentiation potential of iPSCs, or impaired functional activity of iPSC derivatives [189]. Optimization of the reprogramming strategy and culture conditions may contribute in reducing or completely removing such variations [167].

Another important issue is the establishment of quality controls to ensure the safety of human iPSCs and their derivatives designated for downstream application [190]. Serum and mouse-derived feeder cells, used routinely to culture iPS cells, may transmit exogenous antigens or pathogens to reprogrammed cells, causing immune response or disease [191]. Thus, to produce clinical acceptable iPSCs, xeno-free cell culture systems should be used. Several investigations have been conducted and are still in progress, to develop animal-product-free culture system reducing the risks for patients [191]. This can be overcome by developing reliable and reproducible protocols for a direct and efficient differentiation of iPSCs in the desired tissue [192].

Furthermore, pluripotent identity and developmental potential of iPSCs should be characterized by teratoma formation [53]. iPSCs should also be differentiated* in vitro* to test their ability to produce the desired cell type. Evidence shows that epigenetic memories or incomplete reprogramming may affect differentiation properties of iPSCs [131–133], resulting either in a mixed population of differentiated cells or residual undifferentiated cells that may be tumorigenic when transplanted* in vitro*.

Evaluation of iPSC-derived products for cell therapy applications includes preclinical trials in healthy animals and disease models. Rodents are largely used in basic biology of iPSCs; however they may be not predictive of the efficacy. Large animals, such as pigs and monkeys, have been used in preclinical trials [193, 194], since they are more predictive due a more physiological similarity with humans and longer life span. However, there are disadvantages compared to rodents, including higher costs, more complex husbandry, and limited number of disease models.

Toxicity studies should also be performed on iPSC-derived products through the analysis of major organs after transplantation of* in vivo* models [190].

While in USA clinical studies using iPSCs have not received regulatory approval from FDA [187], Japanese regulatory authorities gave the “green light.” A Japanese woman was transplanted with iPSC-derived RPEs to treat macular degeneration [195]. This is the first world pilot study and may be the leader in many other applications of iPSC derivatives.

Cell patient-derived hiPSCs represent a wonderful cellular model to study several genetic diseases. Accordingly, in recent years, a large number of publications have reported that hiPSCs produced from patient with hereditary disease, after differentiation, are able to recapitulate different aspects associated with pathologies [196]. Although cell patient-derived hiPSCs are limited by the fact that they represent a cellular model and they cannot recapitulate all the aspects of a disease and aspects, they remain an extremely valuable tool for the discovery of novel drugs with potential therapeutic applications and for toxicology studies [197]. Using hiPSCs for screening is a more cost-effective strategy than animal testing. In addition, the possibility of producing iPSCs from healthy donors and patients with hereditary or acquired diseases offers a more accurate system to evaluate the exact effect of a drug in a more physiological condition compared to the current cellular models represented by immortalized human cell lines [197]. This advantage combined with their ability to differentiate into a wide range of specialized cells will allow investigators to perform targeted preclinical toxicological* in vitro* trials [197]. Although cell-based* in vitro* assays allow high-throughput and/or high-content screens, they do not reflect the complex scenario* in vivo*.* In vitro* assays must be followed by animal model tests, the only way currently available to obtain a global understanding of crosstalk between different cell types and organs in a living organism, necessary to identify and characterize molecules and to allow their clinical translation from bench to bedside [186]. Moreover, the possibility to produce cell patient-derived iPSCs provided a solution to overcome the strong ethical concerns and immunological rejection that are currently key obstacles to the clinical practice of hESCs.

## 5. Conclusions

It is now indisputable that stem cell research is the way forward to tackle and possibly cure human diseases. PSCs, due to the capacity of differentiating into a wide range cell types, are of most interest where functional adult stem cells types are difficult to access expand or drive. In particular, hiPSCs represent an exciting alternative to embryonic cells, avoiding the ethical issues associated with their use and providing a better model for studying human diseases and possibly finding more effective therapies.

Although the progresses reached so far, further intensive investigations on the properties of human PSCs need to be performed both to understand the basic biology of pluripotency and cellular differentiation and to solve all the different issues associated with therapeutic applications. In addition, improvement of the current technologies should be performed to achieve clinical-grade human PSCs for safe cell therapies.

## Figures and Tables

**Table 1:** Clinical trials using hESC- and hiPSC-derived products (modified by Trounson and McDonald [107]).

Trial sponsor (location)	Targeted disease	Cell type	Phase	Clinical trial ID	Trial status	Reports
Geron Corp. (USA)	Spinal cord injury	hES-derived OPCs	I	NCT01217008	Complete	Not provided

Asterias Biotherapeutics (USA)	Spinal cord injury	hES-derived OPCs	I/IIa	NCT02302157	On-going	Not available yet

Ocata Therapeutics (USA)	Stargardt's macular dystrophy	hES-derived RPE	I/II	NCT01345006	On-going	[108, 109]
	Macular degeneration	hES-derived RPE	I/II	NCT01344993	On-going	
	Myopic macular degeneration	hES-derived RPE	I/II	NCT02122159	Not open yet	—

Pfizer (UK)	Macular degeneration	hES-derived RPE	I	NCT01691261	On-going	Not available yet

Cell Cure Neurosciences Ltd. (Israel)	Macular degeneration	hES-derived RPE	I/IIa	NCT02286089	On-going	Not available yet

Chabiotech Ltd. (South Korea)	Macular degeneration	hES-derived RPEs	I/IIa	NCT01674829	On-going	[110]

ViaCyte (USA)	Type-1 diabetes mellitus	hES-derived PP	I/II	NCT02239354	On-going	Not available yet

Assistance Publique-Hôpitaux de Paris (France)	Severe heart failure	hES-derived CD15 + Isl-1 + progenitors	I	NCT02057900	On-going	Not available yet

International Stem Cell Corp. (Australia)	Parkinson disease	hpESC-derived NSC	I	NCT02452723	Not open yet	—

RIKEN Center for Developmental Biology	Macular degeneration	iPSC-derived RPEs	I	—	On-going	Not available yet
